# The transcription factor Foxg1 regulates telencephalic progenitor proliferation cell autonomously, in part by controlling Pax6 expression levels

**DOI:** 10.1186/1749-8104-6-9

**Published:** 2011-03-18

**Authors:** Martine N Manuel, Ben Martynoga, Mike D Molinek, Jane C Quinn, Corinne Kroemmer, John O Mason, David J Price

**Affiliations:** 1 Genes and Development Group, University of Edinburgh, Hugh Robson Building, George Square, Edinburgh EH8 9XD, UK; 2 National Institute for Medical Research, The Ridgeway, Mill Hill, London NW7 1AA, UK; 3 School of Animal and Veterinary Science, Charles Sturt University, Boorooma Street, Locked Bag 588, Wagga Wagga, NSW 2678, Australia

## Abstract

**Background:**

The transcription factor Foxg1 is an important regulator of telencephalic cell cycles. Its inactivation causes premature lengthening of telencephalic progenitor cell cycles and increased neurogenic divisions, leading to severe hypoplasia of the telencephalon. These proliferation defects could be a secondary consequence of the loss of Foxg1 caused by the abnormal expression of several morphogens (Fibroblast growth factor 8, bone morphogenetic proteins) in the telencephalon of *Foxg1 *null mutants. Here we investigated whether Foxg1 has a cell autonomous role in the regulation of telencephalic progenitor proliferation. We analysed *Foxg1^+/+^*↔*Foxg1^-/- ^*chimeras, in which mutant telencephalic cells have the potential to interact with, and to have any cell non-autonomous defects rescued by, normal wild-type cells.

**Results:**

Our analysis showed that the *Foxg1^-/- ^*cells are under-represented in the chimeric telencephalon and the proportion of them in S-phase is significantly smaller than that of their wild-type neighbours, indicating that their under-representation is caused by a cell autonomous reduction in their proliferation. We then analysed the expression of the cell-cycle regulator Pax6 and found that it is cell-autonomously downregulated in *Foxg1^-/- ^*dorsal telencephalic cells. We went on to show that the introduction into *Foxg1^-/- ^*embryos of a transgene designed to reverse Pax6 expression defects resulted in a partial rescue of the telencephalic progenitor proliferation defects.

**Conclusions:**

We conclude that Foxg1 exerts control over telencephalic progenitor proliferation by cell autonomous mechanisms that include the regulation of Pax6, which itself is known to regulate proliferation cell autonomously in a regional manner.

## Background

Each part of the central nervous system attains a characteristic size during embryogenesis. The vertebrate forebrain (prosencephalon) grows larger than other parts of the neural tube, giving rise to rostral bilateral swellings known as telencephalic vesicles or, when considered together, the telencephalon. The telencephalon is particularly large in mammals and generates the cerebral cortex and basal ganglia. The differential growth of each part of the central nervous system depends critically on region-specific regulation of neural cell proliferation in the embryo. While there is extensive understanding of effector molecules such as cell cycle proteins that control the cell proliferation in general, we know much less about the mechanisms that specify the different rates at which cells divide in each part of the embryo.

The early neural plate and neural tube are patterned by region-specific expression of transcription factors, some of which exert control over cell proliferation as well as other aspects of regional development. For two of these transcription factors, Foxg1 and Pax6, there is strong evidence that one of their primary functions is to regulate telencephalic cell cycles [1-5]. Both are expressed in progenitors in the developing telencephalon, with Foxg1 being activated slightly before Pax6 [4,6,7].

Foxg1 is one of the earliest transcription factors expressed specifically in the part of the neural plate that gives rise to the telencephalon and it remains expressed throughout the telencephalon during embryonic development. Its inactivation leads to severe telencephalic hypoplasia [4]. In a previous study we showed that the cell cycle lengthens prematurely and neurogenic divisions are increased in *Foxg1^-/- ^*telencephalon, thereby reducing the pool of proliferating progenitors [5]. These proliferation defects coincide with reduced expression of the pro-proliferative morphogen Fibroblast growth factor 8 (Fgf8) in the rostral telencephalon and expanded expression of several bone morphogenetic proteins (BMPs), which promote neural differentiation, from their normally dorsally restricted domain [5,8-10]. These observations suggest two possible explanations for the proliferation defects in *Foxg1^-/- ^*telencephalon. First, it is possible that the proliferation defects of *Foxg1^-/- ^*telencephalic progenitors are secondary to abnormal expression of morphogens such as Fgf8 and BMPs. Second, telencephalic progenitors might require Foxg1 cell autonomously for their normal proliferation.

To test whether Foxg1 has a cell autonomous role in the regulation of telencephalic progenitor proliferation, we analysed *Foxg1^+/+^*↔*Foxg1^-/- ^*chimeras, in which mutant telencephalic cells have the potential to interact with, and to have any cell non-autonomous defects rescued by, normal wild-type cells. We found that the *Foxg1^-/- ^*cells were under-represented in the chimeric telencephalon and that the proportion of them in S-phase was significantly smaller than that of their wild-type neighbours, indicating that their under-representation was caused by a cell-autonomous reduction in their proliferation.

We then examined the relationship between Foxg1 and Pax6, in view of their overlapping expression patterns and the fact that Pax6 is known to regulate cell autonomously the proliferation of cortical progenitors [2,3]. We found that Pax6 is cell-autonomously downregulated in *Foxg1^-/- ^*dorsal telencephalic cells. We tested the hypothesis that this contributes to the proliferation defects in *Foxg1^-/- ^*embryos by introducing a transgene designed to reverse Pax6 expression defects into *Foxg1^-/- ^*embryos and found that this partially rescued their telencephalic progenitor proliferation defects.

## Materials and methods

### Animals

Animal care followed institutional guidelines and UK Home Office regulations.

### Derivation of *Foxg1^-/- ^*embryonic stem cells

Wild-type (*Foxg1^+/+^*) or null-mutant (*Foxg1^cre/lacZ^*) embryonic stem (ES) cells were derived using the following protocol. Female mice (129Sv; *Foxg1^+/lacZ^*) were superovulated by intraperitoneal (i.p.) injection of 5U pregnant mare's serum gonadotrophin (Intervet, Milton Keynes, UK) at the middle of the light cycle followed 47 hours later by i.p. injection of 5U human chorionic gonadotrophin (Intervet, UK). Females were mated with *Foxg1^+/cre ^*stud males homozygous for a reiterated *β-globin *repeat transgene (*Tg/Tg*) [11]. Delayed implantation was induced 2.5 days post-coitum by i.p. injection of Tamoxifen (Sigma, Gillingham, UK; 10 μg/animal) and subcutaneous injection of Depo-Provera (Sigma; 1 to 3 mg/animal). At 7.5 days post-coitum, blastocysts were flushed from the uterus, transferred to a gelatinised well containing N2B27 medium with 10 ng/ml leukaemia inhibitory factor and cultured at 37°C in 5% CO_2_. After approximately 5 days, inner cell mass outgrowths were detached from the bottom of each well using a fine pipette and disaggregated in trypsin (0.025% for 2 to 3 minutes at 37°C) to give individual clusters of 1 to 5 cells. Clusters were transferred to a fresh gelatinised well containing N2B27 media containing leukaemia inhibitory factor (10 ng/ml) with the addition of BMP4 (10 ng/ml). Primary ES cell colonies were visible after approximately 5 days in culture. ES cell lines were passaged in feeder-free conditions in BHK-21 Glasgow MEM (GMEM) with 15% fetal bovine serum and leukaemia inhibitory factor (1,000 U/ml). Cell lines were genotyped for presence of *cre*, *lacZ *and *Tg*; their glucose phosphate isomerase 1 (GPI1) isotype was confirmed as described previously [2]. All ES cell lines used for chimera generation were karyotyped and found to have a normal chromosome complement.

### Chimera production and tissue contribution analysis

Chimeras used for tissue contribution and proliferation analyses were produced by injection of *Foxg1^cre/lacZ^;Tg^+ ^*or *Foxg1^+/+^;Tg^+ ^*ES cells into wild-type blastocysts. The ES cells and blastocysts differed at the GPI1 locus. Chimeras were transferred to pseudopregnant females and collected at E12.5. Chimeras were genotyped and the global contribution of ES cell-derived embryonic tissue was estimated using GPI1 electrophoresis as described previously [12]. *Tg^+ ^*cells were visualized in coronal wax sections of the head (8 μm) by DNA-DNA *in situ *hybridisation [13,14]. StereoInvestigator™ (MBF Bioscience, Williston, Vermont, USA) was used to analyse the contribution of *Tg^+ ^*cells: the hippocampus, cortex, ventral telencephalon and dorsal thalamus were delineated and counting boxes of 150 × 150 μm randomly assigned according to the programme's parameters. Three wild-type chimeras (30%, 37%, 40% GPI1A) and three mutant chimeras (12%, 14% and 14% GPI1A) were analysed. Chimeras used for Pax6 expression analysis were produced by the aggregation of preimplantation embryos as described in Manuel *et al.*, 2010. *Foxg1^cre/lacz ^*and *Foxg1^+/lacZ ^*embryos were aggregated with *Foxg1^+/+ ^*embryos to give experimental and control chimeras, respectively. In these cases, the mutant cells were recognizable because the coding sequence of one *Foxg1 *allele is replaced by a *lacZ *reporter cassette [4].

### Elevating Pax6 levels in *Foxg1^-/- ^*embryos

The *Pax77 *transgene [15] was crossed into *Foxg1^-/- ^*embryos to increase their Pax6 levels. This transgene comprises five to seven copies of the human *PAX6 *locus including its upstream and downstream regulatory regions. Our previous studies showed that wild-type mice carrying the *Pax77 *transgene display elevated Pax6 levels at all its sites of expression [3,16].

### Proliferation analyses

Pregnant females at E12.5 were sacrificed 30 minutes after injection with 200 μl of 10 mg/ml bromodeoxyuridine (BrdU; Sigma; in 0.9% NaCl, i.p.). For analysis of proliferation in chimeras, wax coronal sections were immunostained with anti-BrdU following β-globin DNA-DNA *in situ *hybridization. The percentage of *Tg^+ ^*(that is, mutant) progenitors in S-phase was determined by counting the total number of BrdU^+ ^*Tg^+ ^*cells and BrdU^- ^*Tg^+ ^*cells in the ventricular zone of the dorsal and ventral telencephalon of chimeras. To determine the percentage of *Tg*^- ^(that is, wild-type) progenitors in S-phase, BrdU^+ ^*Tg*^- ^and BrdU^- ^*Tg*^- ^cells were counted in 100-μm-wide sampling boxes in the ventricular zone of the dorsal and ventral telencephalon of chimeras.

For analysis of the effects of Pax6 overexpression on proliferation, BrdU^+ ^and BrdU^- ^cells were counted in 100-μm-wide sampling boxes along the ventricular zone of the dorsal telencephalon of wild-type embryos and the whole telencephalon of *Foxg1^-/- ^*and *Foxg1^-/-^;Pax77 *embryos at rostral, central and caudal levels. Each count was repeated on three to five non-adjacent sections from each embryo (n = 3 embryos of each genotype).

### Quantitative reverse transcription-PCR

RNA was extracted from the telencephalon of *Foxg1^-/- ^*and *Foxg1^-/-^;Pax77 *littermates at E12.5 using Qiagen Rneasy kit (Qiagen, Crawley, UK). cDNA synthesis was performed as described in [17]. Analysis of total *Pax6 *levels by quantitative PCR was performed as described in [16].

### Immunohistochemistry

Immunohistochemistry was carried out as described previously [5]. The primary antibodies used were: anti-Pax6 (1:50 to 1:500; Developmental Studies Hybridoma Bank); anti-β-galactosidase (β-gal; 1:800; Invitrogen, Paisley, UK); and anti-BrdU (1:200; Becton Dickinson, Oxford, UK).

### *In situ *hybridisation

Antisense RNA probes for *Emx1 *and *Emx2 *were digoxigenin labelled. *In situ *hybridisations were on 10-μm paraffin sections [18].

## Results

### Foxg1 is required cell-autonomously for normal telencephalic progenitor proliferation

**Figure 1:**
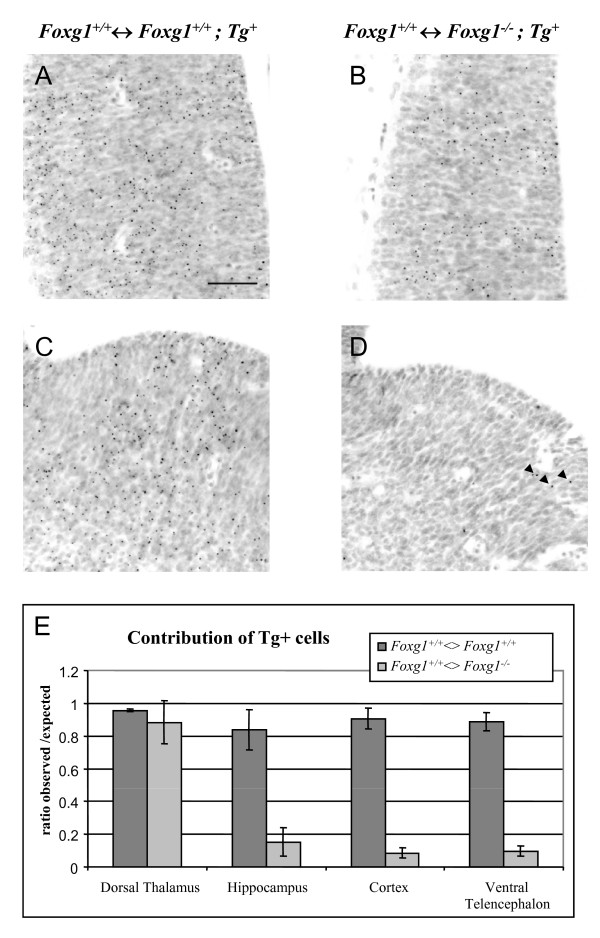
***Foxg1^-/-^*mutant cells are underrepresented in the telencephalon of *Foxg1^+/+^;Tg*^-^↔*Foxg1^-/-^;Tg^+^*chimeras**. **(A-D) **Coronal sections through the thalamus (A,B) and the ventral telencephalon (C,D) of E12.5 *Foxg1^+/+^;Tg*^-^↔*Foxg1^+/+^;Tg^+ ^*control (A,C) and *Foxg1^+/+^*;Tg^-^↔*Foxg1^-/-^;Tg^+ ^*experimental chimeras (B,D) showing *Tg^+ ^*cells (labelled with dark dots) derived from the ES cells. (D) Very few Tg^+ ^cells (arrowheads) are observed in the ventral telencephalon of experimental chimeras. Scale bar: 50 μm. **(E) **Ratios of observed/expected contributions of *Tg^+ ^*cells in the dorsal thalamus, the hippocampus, the cortex and the ventral telencephalon of control (*Foxg1^+/+^;Tg*^-^↔*Foxg1^+/+^;Tg^+^*) and experimental (*Foxg1^+/+^*;Tg^-^↔*Foxg1^-/-^;Tg^+^*) chimeras. *Tg^+ ^*cells are significantly underrepresented in the telencephalon of mutant chimeras (mean ± s.e.m, n = 3 embryos of each genotype; Student's *t*-test, *P *< 0.05).

To test the hypothesis that Foxg1 is required cell autonomously for cells to contribute normally to the telencephalon, we made experimental and control chimeras by injecting *Foxg1^-/- ^*or *Foxg1^+/+ ^*ES cells into wild-type blastocysts. *Foxg1^-/- ^*and *Foxg1^+/+ ^*ES-derived cells carried a *β-globin *transgene (*Tg*) identifiable by DNA *in situ *hybridization, which generated a labelled spot in their nuclei (Figure 1A-D). In each chimeric embryo, an estimate of the percentage of ES-derived cells in Foxg1 non-expressing regions was obtained by quantitative analysis of the GPI1 isozyme composition of the upper body [12]. ES cells produced the GPI1A isozyme, and so the percentage of GPI1 that was GPI1A in the upper body represented the contribution of the ES-derived cells to regions of the embryo where the presence or absence of Foxg1 was predicted to have no effect.

We analysed three experimental and three control chimeras at E12.5 and determined the percentage of *Tg^+ ^*cells in the hippocampus, the cortex and the ventral telencephalon. The dorsal thalamus, in which Foxg1 is not normally expressed, was used as a control brain region. For each of these tissues in each chimera, the observed contribution of *Tg^+ ^*cells (obsTg^+^) was divided by the expected contribution of *Tg^+ ^*cells (expTg^+^) given by the percentage of GPI1A for that chimera. In control chimeras and in the dorsal thalamus of experimental chimeras the obsTg^+^:expTg^+ ^ratios were close to 1, which is the value anticipated in tissues where the contribution of ES-derived cells is no different to that throughout the body of the embryo. *Foxg1^-/- ^Tg^+ ^*cells were present throughout the telencephalon of experimental chimeras, but they were significantly under-represented (Figure 1E). The degree of under-representation was similar in all three tissues studied, that is, the hippocampus, the cortex and the ventral telencephalon.

**Figure 2:**
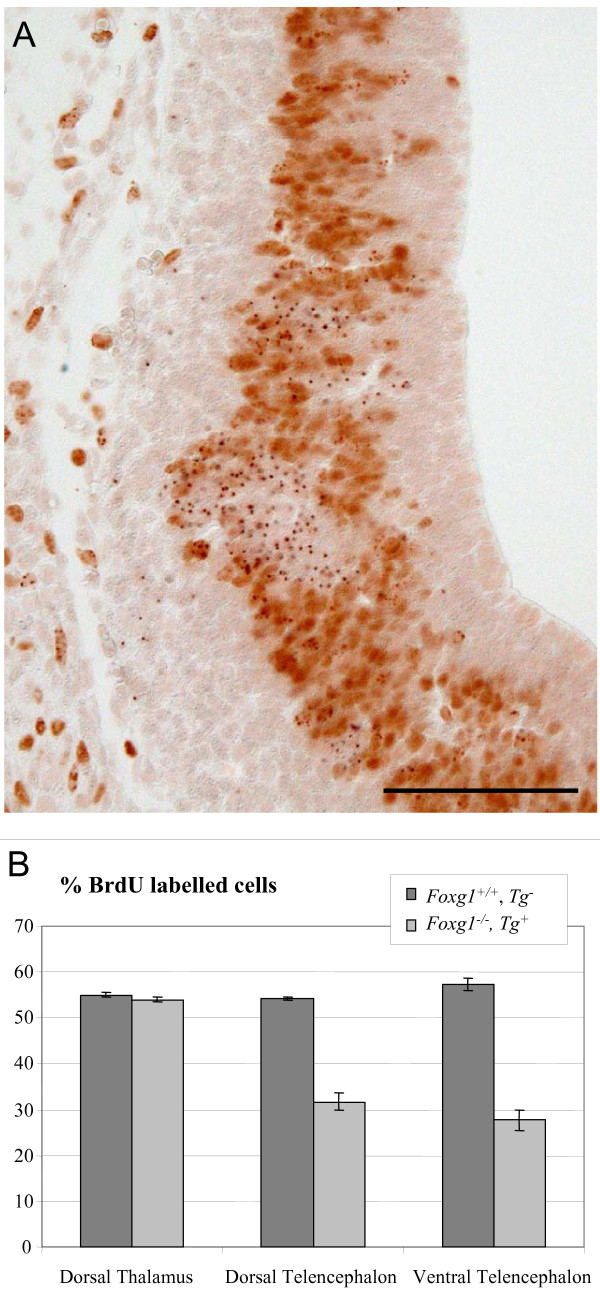
***Foxg1^-/- ^*cells display a cell autonomous proliferation defect**. **(A) **Coronal section through the telencephalon of an E12.5 *Foxg1^+/+^;Tg*^-^↔*Foxg1^-/-^;Tg^+ ^*chimera showing *Tg^+ ^*cells (labelled with dark dots) and BrdU^+ ^cells (with brown nuclei). Clusters of BrdU^- ^*Tg^+ ^*cells are present among BrdU^+ ^*Tg*^- ^and *Tg^+ ^*progenitors. Scale bar: 50 μm. **(B) **Percentages of *Tg^+ ^*cells in S-phase in the dorsal and ventral telencephalon, but not the dorsal thalamus, of experimental chimeras are significantly lower than the percentages of *Tg*^- ^cells in S-phase (mean ± s.e.m, n = 3 embryos of each genotype; Student's *t*-test, *P *< 0.05).

We then tested whether the under-representation of *Foxg1^-/- ^*cells in the telencephalon of chimeras was likely due to a cell autonomous proliferation defect using BrdU to label telencephalic progenitors in S-phase of the cell cycle. In the telencephalic ventricular zone of experimental chimeras, percentages of *Foxg1^-/- ^*cells that were BrdU^+ ^were about half those of surrounding *Foxg1^+/+ ^*cells that were BrdU^+^. The reductions were similar in dorsal and ventral regions (Figure 2A,B). In the dorsal thalamus (Foxg1 non-expressing control tissue), the percentage of *Foxg1^-/- ^*progenitors in S-phase was equal to that of *Foxg1^+/+ ^*progenitors. These results indicate that Foxg1 is required cell autonomously for normal proliferation of telencephalic progenitors.

### Foxg1 is required cell autonomously for normal telencephalic Pax6 expression

**Figure 3:**
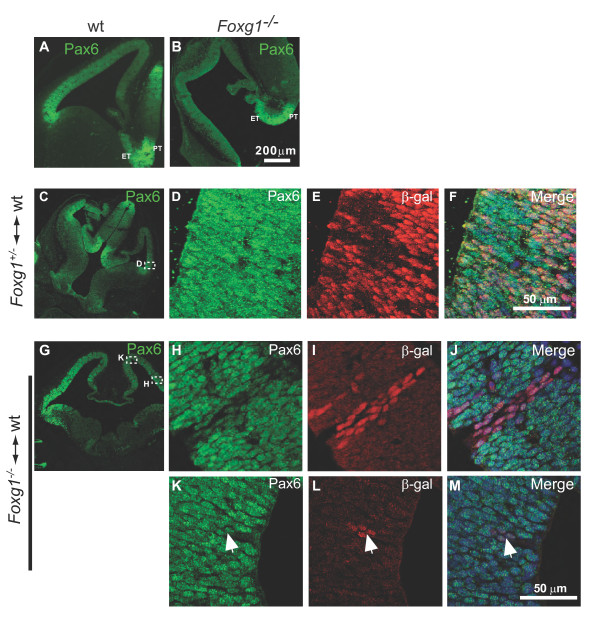
**Pax6 is misregulated in *Foxg1^-/- ^*cells**. **(A,B) **Pax6 immunofluorescence on coronal sections through the telencephalon of wild-type (wt) (A) and *Foxg1^-/- ^*(B) embryos at E12.5. The characteristic lateral^high ^to medial^low ^gradient of Pax6 expression in the telencephalon is observed in wild-type (A) but not in mutant embryos (C). **(C,G) **Pax6 immunofluorescence on coronal sections through the telencephalon of a *Foxg1^+/+^*↔*Foxg1^+/- ^*(C) control chimera and a *Foxg1^+/+^*↔*Foxg1^-/-^*experimental chimera (G). **(D-F,H-J,K-M) **Selected regions are shown at higher magnification and co-labelled for β-gal expressed by *Foxg1^+/- ^*(D-F) or *Foxg1^-/- ^*cells (H-J,K-M). Throughout the whole telencephalon of control chimeras (D-F) and the dorso-medial telencephalon of experimental chimeras (K-M), β-gal^+ ^cells (arrows) express Pax6 at levels indistinguishable from those in adjacent β-gal^- ^cells. In the dorso-lateral telencephalon of experimental chimeras (H-J), however, *Foxg1^-/- ^*(β-gal^+^) cells display markedly lower levels of Pax6 than their neighbouring wild-type (β-gal^-^) cells. ET, eminentia thalami; PT, prethalamus.

We examined the relationship between Foxg1 and the transcription factor Pax6, which is known to regulate telencephalic progenitor proliferation and whose telencephalic expression begins shortly after that of Foxg1 [1-3,6,19,20]. Expression of Pax6 is normally restricted to dorsal telencephalon. In *Foxg1^-/- ^*embryos, progenitors throughout the entire telencephalon express Pax6 [4,5,21] and previous work has shown that this reflects the inability of *Foxg1^-/- ^*telencephalic cells to develop ventral telencephalic fates [21]. Here, we examined the levels of expression of Pax6 in the telencephalon of *Foxg1^-/- ^*embryos and in chimeras. Immunofluorescence for Pax6 showed that, whereas Pax6 is expressed in a rostro-lateral^high ^to caudo-medial^low ^gradient in the dorsal telencephalon in wild-type embryos and control chimeras (Figure 3A,C), in *Foxg1^-/- ^*embryos there is no obvious gradient and Pax6 appears to be expressed at a lower level throughout the telencephalon (Figure 3B). Comparison of Figure 3A and 3B shows a normal level of immunostaining in the *Foxg1^-/- ^*prethalamus and eminentia thalami, a region that does not express Foxg1 and should not be affected in mutants, but comparatively lower immunostaining of the lateral telencephalon.

Regional reduction in immunostaining for Pax6 was very obvious in *Foxg1^-/- ^*cells in *Foxg1^+/+ ^*↔ *Foxg1^-/- ^*chimeras. In rostro-lateral regions of the dorsal telencephalon of experimental chimeras, even very small groups of *Foxg1^-/- ^*cells (recognized by their expression of β-gal) expressed Pax6 at discernibly lower levels than their neighbours (Figure 3H-J). In more medial positions in the dorsal telencephalon of experimental chimeras, where Pax6 levels are lower in *Foxg1^+/+ ^*cells, *Foxg1^-/- ^*cells appeared to express Pax6 at similar levels to their neighbouring *Foxg1^+/+ ^*cells (Figure 3K-M). In control chimeras, levels of Pax6 immunostaining in β-gal-expressing cells and their neighbours were similar (Figure 3D-F). In summary, *Foxg1^-/- ^*cells express Pax6 at low levels, similar to those normally found in caudo-medial telencephalon, through all parts of the telencephalon with no increase in levels in rostro-lateral regions. Our findings from chimeras indicate that the generation of the normal graded increase of Pax6 in the rostro-lateral part of the dorsal telencephalon requires Foxg1 cell-autonomously.

### Pax6 downregulation contributes to the proliferation defects of *Foxg1^-/- ^*telencephalic progenitors

As Pax6 is implicated in the control of cortical progenitor proliferation [1-3,19,20,22,23], we hypothesised that Foxg1 might regulate telencephalic cell proliferation, at least in part, via its regulation of Pax6 levels. The cell autonomous inability of many *Foxg1^-/- ^*dorsal telencephalic cells to achieve normal Pax6 levels might contribute to their proliferation defects. To distinguish between this possibility and an alternative scenario in which loss of Foxg1 prevents normal proliferation independently of any change in Pax6 levels, we generated mice lacking Foxg1 but with elevated Pax6 levels. To do this, we used the Pax6 overexpressing line, Pax77, in which Pax6 levels are elevated within the physiological range in the normal domains of expression of Pax6 [3,15,16].

**Figure 4:**
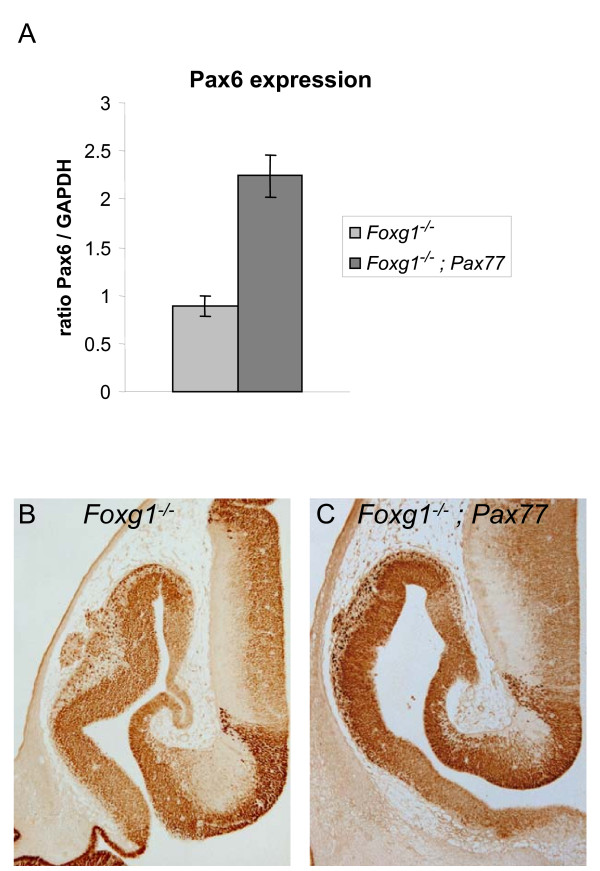
***Pax6 *expression levels are increased in *Foxg1^-/-^;Pax77 *telencephalon compared to *Foxg1^-/-^*telencephalon**. **(A) **Levels of total *Pax6 *mRNA (endogenous *Pax6 *and human *PAX6*) in the telencephalon of E12.5 *Foxg1^-/-^;Pax77 *embryos and *Foxg1^-/- ^*embryos determined by real-time quantitative RT-PCR and normalised against *GAPDH *mRNA levels (mean ± s.e.m, n = 3 in each case). Total *Pax6 *mRNA levels are significantly increased in *Foxg1^-/-^;Pax77 *telencephalon compared to *Foxg1^-/- ^*telencephalon (Student's *t*-test, *P *< 0.05). **(B,C) **Coronal sections through one hemisphere of the forebrain of *Foxg1^-/- ^*(B) and *Foxg1^-/-^;Pax77 *(C) embryos at E12.5, showing the expression of total Pax6 (endogenous Pax6 and human PAX6). Pax6 levels appear increased in *Foxg1^-/-^;Pax77 *compared to *Foxg1^-/- ^*telencephalon, although the lateral^high ^to medial^low ^gradient is not restored.

To confirm that this method successfully increased overall expression in *Foxg1^-/- ^*mutants, we compared the levels of *Pax6 *mRNA in the telencephalon of *Foxg1^-/- ^*and *Foxg1^-/-^;Pax77^+ ^*embryos at E12.5 by quantitative RT-PCR. We found that levels were increased about 2.25-fold in the telencephalon of *Foxg1^-/-^;Pax77^+ ^*embryos compared to *Foxg1^-/- ^*embryos (Figure 4A; Student's *t*-test, *P *< 0.05). This is a similar increase to that found when the *Pax77 *transgene is expressed on a wild-type background [16].

With immunohistochemistry, we observed more intense Pax6 labelling throughout the telencephalon in *Foxg1^-/-^;Pax77^+ ^*embryos (Figure 4C) than in *Foxg1^-/- ^*embryos (Figure 4B). Whereas in *Foxg1^-/- ^*embryos Pax6 immunostaining was much weaker throughout the telencephalon than in the prethalamus and eminentia thalami, in *Foxg1^-/-^;Pax77^+ ^*embryos the intensity of staining in these regions was similar (Figure 4B,C). Pax6 immunolabelling was increased across the telencephalon with no evidence for the restoration of a normal lateral^high ^to medial^low ^(Figure 4B,C) and rostral^high ^to caudal^low ^(not shown) gradient of expression, indicating that the *Pax77 *transgene and the endogenous *Pax6 *locus were being regulated similarly to each other on a *Foxg1^-/- ^*background.

**Figure 5:**
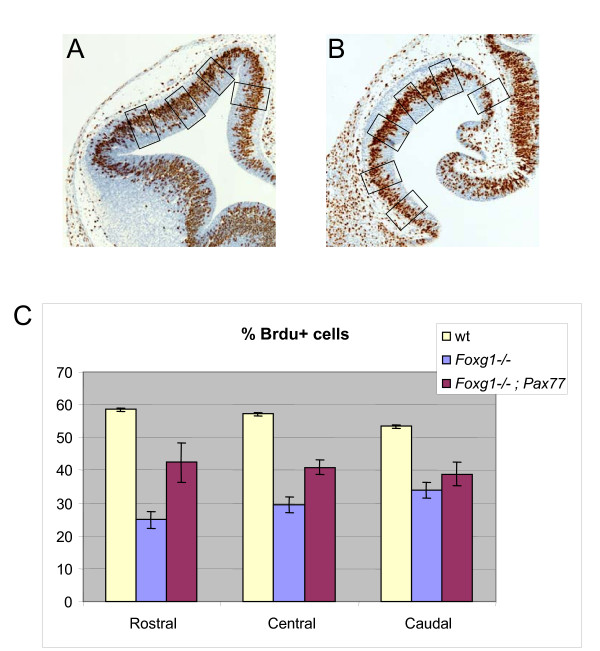
**Proliferation defects are reversed in *Foxg1^-/-^*telencephalon if Pax6 levels are increased**. **(A,B) **Coronal sections through the telencephalon of a wild-type (A) and a *Foxg1^-/- ^*embryo (B) at E12.5 labelled with anti-BrDU (brown). Boxes 100 μm wide show the positions sampled in the wild-type dorsal telencephalon (A) and the whole telencephalon of embryos lacking Foxg1 (B). **(C) **Percentages of cells in S-phase are significantly lower in the *Foxg1^-/- ^*telencephalon compared to the wild-type at rostral, central and caudal levels (mean ± s.e.m. In all three regions: wild-type, n = 3; *Foxg1^-/-^*, n = 4; Student's *t*-test, *P *< 0.01). In *Foxg1^-/-^;Pax77 *embryos, percentages of cells in S-phase are significantly increased compared to those in *Foxg1^-/- ^*embryos at rostral (mean ± s.e.m, n = 4; Student's *t*-test, *P *< 0.05) and central (mean ± s.e.m, n = 4; Student's *t*-test, *P *< 0.01) but not at caudal levels.

As shown in Figures 4B,C, the morphology of the *Foxg1^-/-^;Pax77^+ ^*telencephalon retained its overall resemblance to the *Foxg1^-/- ^*telencephalon at E12.5, with no restoration of ventral telencephalic structure. This is not surprising since previous work has shown that mechanisms independent of Pax6 mediate Foxg1's actions in generating ventral telencephalon [21]. To test whether increased levels of Pax6 countered the proliferation defects in the *Foxg1 *mutant telencephalon, we used BrdU to label telencephalic progenitors in S-phase of the cell cycle. We determined percentages of ventricular zone cells in S-phase (referred to as the labelling indices) in a set of sampling boxes (placed as shown in Figure 5A,B) through the dorsal telencephalon of wild-type embryos (Figure 5A) and through the telencephalon of *Foxg1^-/- ^*and *Foxg1^-/-^;Pax77 *embryos (Figure 5B) at E12.5 at three levels: (i) rostral (normally Pax6^high^): (ii) central; and (iii) caudal (normally Pax6^low^) levels.

Consistent with previous studies [4,5], we found that the average labelling index was significantly lower in the *Foxg1^-/- ^*telencephalon than in the wild-type telencephalon at all three rostro-caudal levels (Figure 5C). At rostral and central levels, average labelling indices were significantly increased in *Foxg1^-/-^;Pax77^+ ^*telencephalon compared to *Foxg1^-/- ^*telencephalon, although they were not restored completely to wild-type levels (Figure 5C). Caudally, the labelling index was not significantly different in *Foxg1^-/-^;Pax77*^+ ^compared to *Foxg1^-/- ^*telencephalon. At rostral, central and caudal levels, labelling indices were similar from dorsal to ventral in the wild-type, *Foxg1^-/- ^*and *Foxg1^-/-^;Pax77*^+ ^telencephalon and in the wild-type dorsal telencephalon and so data from all dorsal to ventral sampling areas were combined for each genotype to generate the histograms in Figure 5C.

We conclude that raising Pax6 levels throughout the telencephalon of *Foxg1^-/- ^*embryos raises proliferation rates in the direction of normal in rostral and central parts of the telencephalon. It does not, however, have a detectable effect in caudal telencephalon.

**Figure 6:**
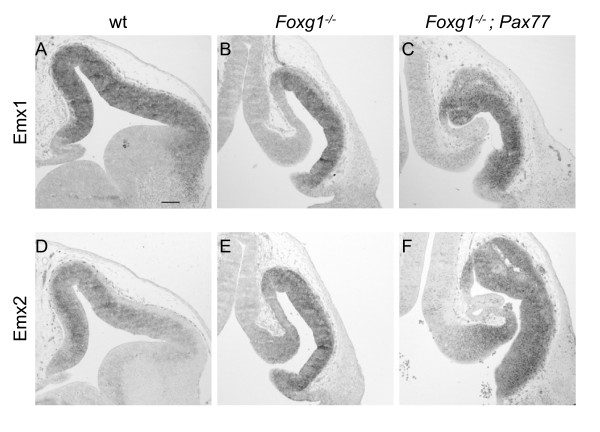
**Increased Pax6 levels do not restore normal *Emx1 *and *Emx2 *expression in the telencephalon of *Foxg1 *mutant embryos**. **(A-F) ***In situ *hybridisation for *Emx1 *(A-C) and *Emx2 *(D-F) on coronal sections through the telencephalon of wild-type (wt) (A,D), *Foxg1^-/- ^*(B,E) and *Foxg1^-/-^;Pax77 *(C,F) E12.5 embryos. Scale bar: 100 μm.

It is possible that Foxg1 and/or Pax6 directly regulate the expression of genes that regulate proliferation, such as cell cycle genes. Alternatively, they might affect proliferation indirectly by controlling the expression of other transcription factors that themselves regulate proliferation. Previous work has shown that Emx1 and Emx2, two transcription factors implicated in the control of cortical progenitor proliferation [24], are misregulated in the *Foxg1^-/- ^*telencephalon [25]. Whereas *Emx1 *and *Emx2 *are expressed in a rostro-lateral^low ^to caudo-medial^high ^gradient in the dorsal telencephalon in wild-type embryos [25] (Figure 6A,D), in *Foxg1^-/- ^*embryos there is no obvious gradient and they appear to be expressed at a high level throughout the telencephalon [25] (Figure 6B,E). Since an earlier study implicated Pax6 as a regulator of *Emx1 *and *Emx2 *expression [26], we wondered whether the observed rise in proliferation rate in the rostral and central telencephalon of *Foxg1^-/-^;Pax77 *embryos might result from a restoration of normal *Emx1 *and *Emx2 *expression. *In situ *hybridisation for *Emx1 *and *Emx2 *expression in the telencephalon of *Foxg1^-/-^;Pax77 *embryos (Figure 6C,F) did not show any obvious change compared to *Foxg1^-/- ^*mutants (Figure 6B,E). It is unlikely, therefore, that elevating Pax6 levels affects proliferation via a change in *Emx1 *or *Emx2 *expression.

## Discussion

Foxg1 and Pax6 are transcription factors essential for early brain development and are implicated particularly strongly in the regulation of telencephalic progenitor proliferation [1-5]. Here we provide evidence linking the activities of the two factors in the regulation of progenitor proliferation. We show that Foxg1 regulates cell autonomously both proliferation and levels of Pax6 expression in telencephalic progenitors. Pax6 is itself already known to regulate telencephalic cell proliferation by cell autonomous mechanisms [2,3]. We show that raising Pax6 levels in *Foxg1^-/- ^*embryos partially reverses their telencephalic proliferation defects. This suggests that reduced proliferation in *Foxg1^-/- ^*telencephalic progenitors can be explained, at least in part, by their reduced Pax6 levels.

Our evidence that Foxg1 regulates cell proliferation cell autonomously is based on data from chimeras in which the proportions of mutant cells are relatively low even in areas that do not normally express Foxg1. The advantage of the mutant cells being greatly outnumbered by the wild-type cells is that it increases the probability of rescuing any cell non-autonomous defects that they might have in *Foxg1^-/- ^*telencephalon, arising, for example, from altered production of intercellular signals such as Fgf8 or BMPs by surrounding cells [5,8-10]. In chimeras, the labelling indices of mutant telencephalic cells (that is, the percentages of mutant cells in S-phase of the cell cycle) were around 30% (Figure 2B), which is the same as the labelling indices of mutant cells in full *Foxg1^-/- ^*mutants (Figure 5C). This means that the proliferative defects of *Foxg1^-/- ^*cells might be accounted for entirely by cell autonomous defects, but it does not exclude the possibility that cell non-autonomous proliferation-enhancing processes such as intercellular signalling are defective in full *Foxg1^-/- ^*mutants.

When examined in detail, the relationship between Foxg1 and Pax6 is not straightforward. Interestingly, while loss of Foxg1 lowers overall Pax6 expression in the telencephalon, the magnitude of the effect is regional: the greatest reduction is in those areas where Pax6 expression is normally highest, that is, rostro-laterally. The consequence is to abolish the normal gradient of expression of Pax6 across the telencephalon. Since in normal telencephalon Foxg1 expression levels are not linearly related to Pax6 expression levels - for example, Foxg1 is normally expressed in some ventral regions where Pax6 is not [4,7] - it seems most likely that Foxg1 is an essential requirement for activation of normal telencephalic Pax6 expression in combination with additional factors. Together these factors might activate Pax6 expression and raise its levels rostro-laterally; Foxg1 is a required component in this process and its loss causes Pax6 expression to fall to basal levels normally found caudo-medially.

The ideal rescue experiment would have involved reactivation of the graded expression of Pax6 across the telencephalon in a *Foxg1^-/- ^*embryo. This is, however, not feasible with existing tools. Our approach increased Pax6 levels in *Foxg1^-/- ^*telencephalon in a controlled manner within a physiological range but did not restore the gradient of expression. Immunohistochemistry suggested that levels were raised throughout the telencephalon to those normally seen in the lateral telencephalon, prethalamus and eminentia thalami. Interestingly, while this raised overall proliferation rates in the *Foxg1^-/- ^*telencephalon, effects were again regional with the greatest rescue seen rostrally, coinciding with the region where Pax6 is normally highest [7].

Previous studies have shown that normal levels of Pax6 are particularly important for regulating proliferation in the rostral part of the telencephalon where Pax6 levels are normally highest [3,19]. The simplest explanation for the failure of caudal telencephalic progenitors to increase their proliferation in response to elevation of Pax6 levels is that they are not competent to respond to this increase and their proliferation is regulated mainly by Foxg1-dependent factors other than Pax6.

Even rostrally, elevation of Pax6 levels in *Foxg1^-/- ^*telencephalon did not restore normal proliferation. There are several possible explanations for this. Probably the best is that Foxg1 regulates telencephalic progenitor proliferation through pathways that do not involve Pax6 as well as through pathways that do involve Pax6. The Pax6-independent pathways might be cell autonomous or cell non-autonomous. While our chimera experiments provide clear evidence that Foxg1 regulates cell proliferation cell autonomously, they do not exclude the possibility of cell non-autonomous defects with the potential to influence telencephalic progenitor proliferation in *Foxg1^-/- ^*embryos. It is known, for example, that *Foxg1^-/- ^*embryos have reduced expression of the pro-proliferative intercellular signalling molecule Fgf8 [5]. Cell autonomous actions of Foxg1 might include direct regulation of the transcription of cell cycle genes in telencephalic progenitors but there is currently little evidence on which to base strong hypotheses. For example, while previous studies have shown that Foxg1 can inhibit TGF-beta-mediated anti-proliferative responses through suppressing *p21 *transcription and P21 is expressed in an expanded domain in *Foxg1 *mutants, we have shown previously that P21 is not upregulated in *Foxg1^-/- ^*telencephalic cells in chimeras [21].

## Conclusions

In their original description of the functions of Foxg1, Xuan *et al. *[4] described a major proliferation defect as the most prominent feature of the *Foxg1^-/- ^*phenotype. Subsequent work has reinforced this conclusion and has added important information on the importance of Foxg1 for normal development of telencephalic dorso-ventral structures [5,21,27]. Here we focussed on the gene's pro-proliferative function. We conclude that Foxg1 exerts control over telencephalic progenitor proliferation by cell autonomous mechanisms that include the regulation of Pax6, which itself regulates proliferation cell autonomously in a regional manner.

## Abbreviations

β-gal: β-galactosidase; BMP: bone morphogenetic protein; BrdU: bromodeoxyuridine; ES: embryonic stem; Fgf: Fibroblast growth factor; GPI: glucose phosphate isomerase; i.p.: intraperitoneal.

## Competing interests

The authors declare that they have no competing interests.

## Authors' contributions

MNM participated in design and supervision, carried out some of the experiments and co-wrote the paper. BM, JCQ and MDM designed and carried out some of the experiments. CK carried out some of the experiments. JOM participated in design, supervision and analysis. DJP participated in design and supervision and co-wrote the paper.
